# Transcranial low-intensity pulsed ultrasound in neurological disorders: mechanisms, therapeutic applications, and translational challenges

**DOI:** 10.3389/fneur.2026.1850924

**Published:** 2026-07-14

**Authors:** Hongbo Cai, Yazhe Wang, Shuhan Che, Zhitao Hou

**Affiliations:** 1 The First Affiliated Hospital of Heilongjiang University of Chinese Medicine, Harbin, Heilongjiang, China; 2 Institute of Shaanxi Key Laboratory of Ultrasonics, Shaanxi Normal University, Xi’an, China; 3 College of Basic Medical and Sciences, Heilongjiang University of Chinese Medicine, Harbin, Heilongjiang, China

**Keywords:** brain repair, low-intensity pulsed ultrasound, neuroinflammation, neurological disorders, neuromodulation

## Abstract

Transcranial low-intensity pulsed ultrasound (LIPUS) is an emerging non-invasive modality with high spatial precision, substantial tissue penetrability, and favorable biosafety. Acting predominantly through mechanical rather than thermal bioeffects, LIPUS modulates mechanosensitive ion channels, intracellular calcium signaling, synaptic transmission, glial activation, neurovascular coupling, and, in selected settings, blood–brain barrier permeability. These features support its growing application in neurological disorders. In this review, we summarize the mechanistic basis of transcranial LIPUS and discuss its therapeutic applications in Alzheimer’s disease, Parkinson’s disease, epilepsy, ischemic stroke, and major depressive disorder. Across these conditions, LIPUS has been associated with neuroprotection, enhanced synaptic plasticity, suppression of pathological neural activity, attenuation of neuroinflammation, promotion of vascular remodeling, and facilitation of targeted delivery through reversible blood–brain barrier opening. We further highlight the major barriers to clinical translation, including heterogeneity of stimulation parameters, incomplete mechanistic understanding, limited comparability across studies, and insufficient large-scale clinical validation. Current evidence supports transcranial LIPUS as a promising ultrasound-based platform for neuromodulation and brain repair, while emphasizing the need for standardized protocols and rigorous translational studies.

## 1 Biophysical basis and mechanisms of transcranial LIPUS

**Figure 1:**
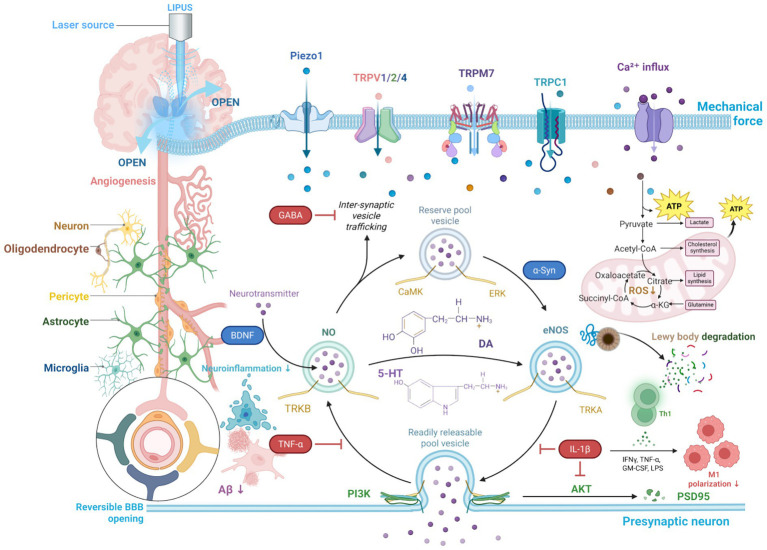
Multi-scale mechanistic framework of transcranial low-intensity pulsed ultrasound (LIPUS) in neurological disorders. Transcranial LIPUS non-invasively delivers pulsed acoustic energy through the intact skull to targeted cortical or deep brain regions, where it exerts predominantly mechanical rather than thermal bioeffects. (1) As illustrated on the left side of the figure, these effects may occur at the system and tissue levels, including reversible blood–brain barrier (BBB) opening, angiogenesis, modulation of neurovascular unit components, and regulation of multiple brain-resident cell types such as neurons, astrocytes, oligodendrocytes, pericytes, and microglia. These system-level responses provide a structural and microenvironmental basis for ultrasound-mediated neuromodulation and neurorepair. (2) At the membrane level (upper central panel), mechanical perturbation induced by LIPUS is proposed to activate mechanosensitive pathways and ion channels, including Piezo1, TRPV-family channels, TRPM7, and TRPC1, leading to calcium influx and altered membrane excitability. These early biophysical events are considered upstream triggers for subsequent intracellular and synaptic responses. Because the precise contribution of individual channels may vary across experimental systems, the channels shown here should be interpreted as representative candidate mediators rather than universally established obligatory effectors. (3) At the synaptic and intracellular levels (central and right panels), LIPUS-associated signaling is linked to modulation of neurotransmitter release, reserve-pool and readily releasable vesicle dynamics, and activation of downstream pathways involving CaMK, ERK, PI3K/AKT, TRKB, eNOS, and nitric oxide signaling. These responses may influence neurotrophic support, synaptic plasticity, mitochondrial metabolism, oxidative stress, and inflammatory signaling. The figure also summarizes reported associations with reduced neuroinflammatory mediators, altered microglial polarization, promotion of PSD95-related synaptic integrity, and regulation of disease-relevant molecules such as *α*-synuclein, although not all of these mechanisms have been demonstrated in every disease model. (4) At the disease level, the convergence of these multi-scale responses may contribute to circuit remodeling, neuroprotection, vascular repair, and functional recovery across neurological disorders, including Alzheimer’s disease, Parkinson’s disease, epilepsy, ischemic stroke, and major depressive disorder. This figure is intended as a conceptual summary of current evidence and proposed mechanisms and does not imply that all pathways occur simultaneously or equivalently in every experimental or clinical setting.

Low-intensity pulsed ultrasound (LIPUS) is an emerging non-invasive neuromodulatory modality distinguished by its use of low-intensity (<3 W/cm^2^) pulsed ultrasound output. This technology confers distinct advantages, including millimeter-scale spatial resolution for highly precise neural modulation and outstanding tissue penetrability, which permits noninvasive transmission through the intact skull to depths greater than 10 cm, enabling targeted regulation of cortical as well as deep brain structures (1). Relative to high-intensity focused ultrasound, LIPUS exhibits a more favorable safety profile, characterized by reversible neuromodulatory actions, a reduced propensity to induce tissue standing waves, and minimal thermal deposition. These unique physical properties make LIPUS an ideal tool for research on neurological disorders, and it has already shown therapeutic potential in a variety of conditions, including Alzheimer’s disease (AD), Parkinson’s disease (PD), epilepsy, ischemic stroke, and major depressive disorder (MDD). The biological effects of LIPUS are driven predominantly by mechanical mechanisms, whereby high-frequency sound waves (usually 0.5–5.0 MHz) traveling through tissue produce periodic compression and rarefaction, and these mechanical perturbations selectively activate membrane mechanosensitive channels (MSCs). As key mediators of mechanochemical signal transduction, MSCs undergo conformational changes under ultrasonic stimulation, thereby increasing their probability of opening. Yoo et al. (2) demonstrated that ultrasound significantly elevates neuronal intracellular Ca2 + concentrations, while pharmacological inhibition of MSCs markedly diminishes neuronal responses, suggesting that MSCs serve as critical mediators of ultrasound-evoked calcium signaling in neurons. Ultrasound may also modulate membrane excitability and downstream synaptic activity, thereby influencing neurotransmitter release and central synaptic transmission efficiency. In addition to the experimentally supported role of mechanically induced calcium signaling, Piezo channels and members of the transient receptor potential (TRP) family have been proposed as candidate mediators of ultrasonic mechanotransduction in neural systems (1, 3). However, the precise contribution of individual channel subtypes remains context-dependent and has not yet been uniformly established across different experimental models. These observations provide important insight into the mechanistic basis of ultrasonic neuromodulation and support further exploration of LIPUS as a tool for neural plasticity and neurorepair (Figure 1). This review systematically summarizes recent progress in the application of transcranial LIPUS to neurological diseases, including ischemic stroke, PD, AD, epilepsy, and MDD.

## 2 Therapeutic applications of transcranial LIPUS in Alzheimer’s disease

Alzheimer’s disease (AD), one of the major neurodegenerative disorders, is characterized by extracellular amyloid-*β* deposition, intracellular tau pathology, and progressive synaptic and neuronal loss, particularly in memory-related brain networks (4). Therapeutic development is further constrained by the blood–brain barrier (BBB), which severely limits brain delivery of most small-molecule drugs and nearly all large-molecule therapeutics (5). In this context, LIPUS exhibits promising therapeutic potential, and its mechanisms may be systematically summarized in three principal aspects. First, reversible BBB modulation represents an important translational opportunity. Through ultrasound-mediated cavitation in the presence of microbubbles, transient and spatially localized BBB opening can be achieved, thereby creating new possibilities for targeted therapeutic delivery. In a translational human study, Mehta et al. (6) reported MRI-guided focused ultrasound-induced BBB permeabilization in patients with early AD, supporting the feasibility of localized and reversible ultrasound-mediated BBB modulation in the clinical setting. In addition, He et al. (7) showed that BBB-crossing delivery of felodipine ameliorated anxiety-like behavior and cognitive impairment in an AD model, further supporting the therapeutic relevance of ultrasound-enabled delivery strategies. Second, LIPUS exerts multitarget neuroprotective effects in neuromodulation. It may facilitate angiogenesis and myelin-related repair. Eguchi et al. (8) showed that whole-brain LIPUS stimulation improved the neural microenvironment through upregulation of vascular endothelial-associated genes and promotion of oligodendrocyte proliferation, with endothelial nitric oxide synthase (eNOS) serving as an important mediator. Intermittent LIPUS treatment significantly improved cognitive performance in dementia model mice, and the beneficial effects persisted for at least 3 months. In addition, LIPUS may enhance synaptic plasticity. Wang et al. (9) reported that LIPUS was associated with increased expression of hippocampal PSD95 and synaptophysin, which may contribute to improvements in learning and memory function. Moreover, LIPUS may suppress neuroinflammatory responses. Tramontin et al. (10) showed that LIPUS reduced astrocytic activation, attenuated pro-inflammatory signaling, and decreased amyloid-related pathological burden, thereby helping to interrupt the self-perpetuating cycle of neuroinflammation and neuronal injury. Third, available evidence supports a favorable preliminary biosafety and clinical feasibility profile. Beisteiner et al. (11) conducted a clinical study in 35 patients with AD who received a 2-week course of transcranial pulse stimulation, with cognitive improvement maintained for up to 3 months after treatment; imaging examinations, including MRI, together with clinical monitoring, revealed no treatment-related cerebral hemorrhage, cerebral edema, or other newly developed intracranial lesions, suggesting a favorable short-term safety profile. Subsequent human studies further support the clinical feasibility of ultrasound-based intervention in AD, while also indicating that the field remains at an early and heterogeneous stage of development. In particular, current clinical translation is proceeding along two partially convergent paths: a neuromodulatory route (including TPS, LIPUS, and LIFU) and a BBB-opening/drug-delivery route based on focused ultrasound with microbubbles. Notably, a multicenter, randomized, double-blind, placebo-controlled pivotal/phase III trial of whole-brain LIPUS in early AD is ongoing in Japan (LIPUS-AD; NCT05983575), with change in ADAS-J-cog-14 from baseline to 72 weeks as the primary endpoint. By contrast, most other clinical studies in this field, including focused ultrasound-mediated BBB opening and early neuromodulation trials, remain at the level of safety, feasibility, or proof-of-concept evaluation. Thus, although ultrasound-based intervention shows clear promise in AD by enabling transient BBB modulation to support targeted delivery while simultaneously exerting neuroprotective and neuromodulatory effects, it should currently be regarded as a promising but still investigational adjunctive strategy rather than an established component of integrated AD therapy.

## 3 Therapeutic applications of transcranial LIPUS in Parkinson’s disease

As the second most common neurodegenerative disease, PD is pathologically characterized by the progressive degeneration of dopaminergic neurons in the substantia nigra pars compacta of the midbrain, and by the time typical motor symptoms emerge, neuronal loss often reaches 50–70% (12). Pathological investigations (13) have delineated four central mechanisms in PD: aberrant *α*-synuclein aggregation leading to Lewy body formation, oxidative stress triggered by mitochondrial complex I dysfunction, loss of proteostasis caused by ubiquitin–proteasome system dysregulation, and neuroinflammation-driven deterioration of the neural microenvironment. LIPUS has demonstrated distinct modulatory effects across all four of these core mechanisms: (1) In terms of neurotrophic factor regulation, LIPUS can stimulate intact subcortical brain circuits in deep regions of the mouse brain and increase the expression of endogenous brain-derived neurotrophic factor (BDNF) (14); LIPUS may also directly target astrocytes, markedly enhancing BDNF protein expression in a time-dependent fashion through activation of the TrkB/PI3K/Akt, Ca2+/CaMK, and NF-κB signaling pathways, thereby modulating the development and function of neural circuits (15). (2) Regarding the modulation of oxidative stress, LIPUS primarily acts by attenuating reactive oxygen species-induced neuronal damage, with the underlying mechanisms including: First, Antioxidant regulation and improvement of mitochondrial function. Zhou et al. (16) reported that LIPUS lowers oxidative stress levels in the brains of PD mice, suppresses reactive oxygen species generation, and mitigates mitochondrial dysfunction, thereby protecting dopaminergic neurons against free radical-induced injury, likely through modulation of antioxidant proteins and enhancement of mitochondrial complex I activity; Second, Preservation of mitochondrial energy metabolism. LIPUS inhibits the reduction in mitochondrial membrane potential in dopaminergic neurons, preserves normal mitochondrial ATP generation, and reduces reactive oxygen species accumulation, thereby decreasing the loss and apoptotic death of dopaminergic neurons in the substantia nigra of PD model mice (17). (3) Neuroinflammatory regulation is critically important, because a microglia-dominated inflammatory microenvironment represents a major barrier to effective PD therapy. Research (18) has demonstrated that LIPUS inhibits proinflammatory microglial responses in the brain by decreasing the expression of tumor necrosis factor-*α* (TNF-α) and interleukin-1β (IL-1β) in dopaminergic neurons. Following microelectrode implantation, LIPUS treatment effectively alleviates the inflammatory cascade and foreign body response induced by microglial activation, inhibits M1 polarization of microglia in the mouse brain, and enhances their phagocytic function (19).

Importantly, recent studies in human clinical populations further indicate that ultrasound-based neuromodulation is moving beyond preclinical proof-of-concept in PD. As highlighted by recent reports, transcranial ultrasound approaches have been explored for individualized non-invasive targeting of basal ganglia circuits and for the suppression of pathological oscillatory activity in patients with Parkinson’s disease (20, 21). Although these human data remain early-stage and should be interpreted cautiously, they provide an important translational complement to the preclinical literature and suggest that ultrasound-based intervention in PD may have growing clinical relevance. In summary, LIPUS provides a novel strategy for PD treatment through a triadic mechanism of “neurotrophic support–oxidative balance–inflammatory regulation,” and its advantages, including precise targeting of deep brain regions (with a localization error of <1 mm) and noninvasiveness, make it particularly suitable for chronic disorders such as PD that require long-term intervention. Future studies should focus on resolving issues related to the standardization of treatment parameters, deeper elucidation of mechanisms of action, and clinical translation.

## 4 Therapeutic applications of transcranial LIPUS in epilepsy

As a chronic disorder of the central nervous system, epilepsy is pathologically characterized by abnormal hypersynchronous discharges of neuronal populations in the brain, resulting in recurrent episodes of impaired consciousness. In existing treatment paradigms, antiepileptic medications, including carbamazepine and valproate, are limited by suboptimal efficacy and prominent side effects, while surgical treatment is often restricted by challenges in accurately localizing epileptogenic zones, especially in insular or multifocal epilepsy, highlighting the pressing need for new neuromodulatory approaches. By virtue of its high spatiotemporal precision, LIPUS exhibits distinct advantages in epilepsy treatment, which are manifested in the following respects: (1) Inhibition of epileptic activity: Lin et al. (22) found that after 30 min of LIPUS treatment in epileptic monkey models, both the total number of seizures and the hourly seizure frequency were significantly reduced within 8 h, with an inhibition efficiency exceeding 65%; moreover, LIPUS not only suppresses acute epileptic activity and status epilepticus, but also reduces seizure frequency in recurrent epilepsy, with the average daily seizure frequency falling to less than one episode after treatment (23). (2) Modulation of neural oscillations: an experimental study using a mouse model of epilepsy (24) showed that LIPUS markedly decreased low-frequency (<10 Hz) local field potential intensity, while concurrently modulating slow and fast neural oscillations, thereby effectively disrupting epileptiform synchronized discharge rhythms; this intervention not only significantly suppressed epileptiform discharges but also prolonged the interictal interval by nearly fourfold, suggesting a sustained antiepileptic effect. These findings indicate that LIPUS exerts a pronounced neuromodulatory effect in the treatment of epilepsy. Mechanistic analysis by Chen et al. (25) revealed that LIPUS exerts therapeutic effects by inhibiting the PI3K-Akt–mTOR signaling pathway: first, it decreases the amplitude of cortical local field potentials; second, it modulates neuronal activity via a dual mechanism, simultaneously suppressing excitatory neurons and activating GABAergic terminals, thereby effectively restraining abnormal cortical discharges. These findings provide important theoretical support for the application of LIPUS in epilepsy treatment. In summary, LIPUS exhibits significant antiepileptic effects by suppressing epileptiform discharges and modulating neural oscillations, and its noninvasive and precise regulatory properties offer a new direction for epilepsy treatment. Future studies should further optimize stimulation parameters, investigate long-term efficacy, and promote clinical translation.

## 5 Therapeutic applications of transcranial LIPUS in ischemic stroke

Among cerebrovascular disorders, ischemic stroke represents a major global public health challenge owing to its high incidence, high disability, and high recurrence. In recent years, transcranial low-intensity ultrasound–based neuromodulation, including LIPUS and closely related focused ultrasound paradigms, has emerged as a promising non-invasive strategy for stroke rehabilitation. Current evidence suggests that its therapeutic effects are mainly reflected in three aspects. First, in terms of neural repair, stroke-specific studies indicate that ultrasound promotes post-stroke angio-neurogenesis and white-matter repair rather than the previously cited unsupported pathways. In a mouse MCAO model, Ichijo et al. (26) reported that whole-brain LIPUS improved tightrope and rotarod performance at 28 days and increased CD31-positive vessels in the perifocal lesion and doublecortin-positive neurons in the ischemic striatum; these benefits were absent in eNOS-deficient mice, supporting an eNOS-dependent angio-neurogenic mechanism. The same study also showed upregulation of VEGF, SDF-1, CXCR4, and BDNF, suggesting activation of migration- and neurotrophin-related repair programs. More recently, Wang et al. (27) showed that LIPUS protected white matter integrity, reduced early neuronal and oligodendrocyte progenitor cell death, and promoted oligodendrocyte maturation and remyelination after stroke, possibly through downregulation of the IL-17A/Notch1 pathway. Second, in vascular remodeling, Guo et al. (28) found that pulsed transcranial ultrasound applied immediately after distal MCAO significantly increased cerebral blood flow, reduced neutrophil infiltration, and decreased infarct volume at 48 h. Consistently, Chen et al. (29) reported that LIPUS initiated 0.5 h after ischemia increased cerebral blood flow, reduced brain swelling, and promoted leptomeningeal vascular remodeling, including greater vessel diameter as well as increased vessel length and density, with inhibition of ROCK1/p-MLC2 signaling implicated as a potential mechanism. Third, against secondary brain injury, ultrasound-based stimulation appears to exert anti-apoptotic and blood–brain barrier–protective effects. Chen et al. (30) demonstrated that LIPUS pretreatment reduced neuronal apoptosis and restored BDNF expression in MCAO mice, and Wu et al. (31) further showed reduced neuronal apoptosis, lower mortality, and improved neurological outcomes in a recurrent stroke model. In addition, Deng et al. (32) reported that transcranial focused ultrasound attenuated vasogenic edema and BBB disruption after MCAO, preserved ZO-1 expression, reduced IgG leakage, and suppressed TNF-*α* and MMP-9 in the ischemic brain. Overall, current evidence supports a multimodal role for transcranial low-intensity ultrasound in ischemic stroke, involving neurorestoration, vascular remodeling, and mitigation of secondary injury. Nevertheless, clinical validation remains limited, and rigorously designed studies are still required to define optimal stimulation parameters, therapeutic windows, and long-term safety.

## 6 Therapeutic applications of transcranial LIPUS in major depressive disorder

In recent years, the incidence of MDD has increased year by year, with an annual prevalence of 2.1% (33), making it one of the major psychiatric disorders in China and worldwide, and it is currently believed to be associated with neurotransmitter imbalance and abnormal serotonergic function in the brain. LIPUS enables precise, non-invasive regulation of targeted brain regions, with the capacity to modulate serotonin levels, enhance synaptic plasticity, and exert additional effects such as reducing neuroinflammation and regulating BBB permeability.

(1) Regulation of neurotransmitter systems: Zhu et al. (34) showed that LIPUS stimulation of the dorsal raphe nucleus in mice effectively prevents aberrant serotonin depletion and significantly ameliorates depression-like behaviors, manifested by increased sucrose preference and shortened immobility time in the tail suspension test; these findings indicate that LIPUS exerts a significant modulatory effect on central neurotransmitter systems. Further research (35) found that LIPUS intervention applied to the thalamic region in a rat model of MDD not only increases local extracellular serotonin concentration but also, through neural circuit modulation, synchronously elevates dopamine levels in the downstream medial prefrontal cortex. These studies collectively reveal a potential mechanism by which LIPUS regulates the monoaminergic neurotransmitter system through multiple targets. (2) Enhancement of synaptic plasticity: One study (36) found that following LIPUS intervention targeting the medial prefrontal cortex or ventromedial prefrontal cortex, MDD model rats showed not only behavioral improvement but also a marked increase in dendritic spine density of cortical neurons, together with significant upregulation of key synaptic proteins, including NR2B, PSD95, and SYP. Further mechanistic investigation (37) suggests that the observed enhancement of synaptic plasticity is closely linked to activation of the BDNF/ERK/mTORC1 signaling axis in the prefrontal cortex by LIPUS; moreover, 4 weeks of LIPUS treatment was found to be both safe and effective and significantly increased the number of c-Fos-positive cells in the ventromedial prefrontal cortex, suggesting that LIPUS can rapidly enhance neuronal activity in the prefrontal cortex. These studies provide theoretical evidence that LIPUS exerts antidepressant effects through modulation of synaptic plasticity. (3) Anti-inflammatory effects and BBB-opening mechanisms: In the treatment of MDD, LIPUS demonstrates distinct advantages in anti-inflammatory modulation and targeted drug delivery. Yi et al. (38) confirmed that LIPUS markedly ameliorates depression-like behaviors in lipopolysaccharide-induced mouse models of MDD, potentially through suppression of key proinflammatory mediators, including IL-6, IL-1β, and TNF-*α*, in the prefrontal cortex. As in other neurological disorders, the presence of the BBB severely limits the efficiency of central delivery for most antidepressant therapeutics. When combined with microbubble technology, LIPUS can transiently and controllably open the BBB with high precision, thereby enabling targeted delivery of neurotrophic factors such as glial cell line-derived neurotrophic factor, significantly increasing serotonergic neuron numbers, and upregulating tryptophan hydroxylase 2 expression. This multitarget intervention strategy provides a new technological approach for the treatment of MDD.

## 7 Translational challenges and future perspectives

In summary, transcranial low-intensity pulsed ultrasound (LIPUS) has emerged as a highly promising modality for neurological intervention, distinguished by its non-invasive nature, high spatial precision, deep tissue penetrability, and multimodal biological effects. As discussed above, its therapeutic potential extends beyond conventional neuromodulation, encompassing regulation of neuronal excitability, synaptic plasticity, neuroinflammation, oxidative stress, neurovascular coupling, and, in selected contexts, blood–brain barrier permeability (Table 1). These convergent actions position LIPUS not merely as a stimulation tool, but as a potentially versatile platform for circuit modulation, tissue repair, and targeted therapeutic delivery across a wide range of neurological disorders.

**Table 1:** Transcranial LIPUS across major neurological disorders.

Disorder	Core pathological barrier	Principal LIPUS actions	Key therapeutic implications	Representative references
Alzheimer’s disease (AD)	1. Amyloid-*β* deposition 2. Tau pathology 3. Synaptic and neuronal loss 4. BBB-limited therapeutic delivery	1. Transient and localized BBB modulation 2. Promotion of angiogenesis and myelin-related repair 3. Enhancement of synaptic plasticity 4. Suppression of neuroinflammatory signaling 5. Early clinical feasibility of ultrasound-based intervention	1. Supports targeted delivery to the brain 2. Improves cognitive function in preclinical models 3. Suggests short-term clinical feasibility 4. Remains a promising but investigational adjunctive strategy	(4–11)
Parkinson’s disease (PD)	1. Degeneration of nigrostriatal dopaminergic neurons 2. α-Synuclein pathology 3. Oxidative stress and mitochondrial dysfunction 4. Neuroinflammation	1. Upregulation of neurotrophic support (e.g., BDNF) 2. Reduction of oxidative stress 3. Preservation of mitochondrial function 4. Inhibition of microglial activation 5. Emerging circuit-level modulation in human studies	1. Protects dopaminergic neurons in preclinical models 2. Supports chronic circuit preservation 3. Provides early translational evidence for non-invasive basal ganglia modulation in humans	(12–21)
Epilepsy	1. Pathological hypersynchronous neuronal firing 2. Limited drug responsiveness 3. Difficulty localizing epileptogenic foci	1. Suppression of epileptiform discharges 2. Modulation of pathological neural oscillations 3. Inhibition of PI3K–Akt–mTOR signaling 4. Enhancement of inhibitory tone	1. Reduces seizure burden 2. Disrupts epileptic network synchronization 3. Supports a non-invasive neuromodulatory strategy for seizure control	(22–25)
Ischemic stroke	1. Neuronal loss and incomplete functional recovery 2. Impaired neurovascular remodeling 3. White-matter injury and secondary BBB disruption	1. Promotion of post-stroke angio-neurogenesis 2. Enhancement of remyelination and white-matter repair 3. Improvement of cerebral blood flow and vascular remodeling 4. Reduction of apoptosis, BBB disruption, and vasogenic edema	1. Supports neurological recovery after stroke 2. Coordinates neurorestoration and vascular repair 3. Mitigates secondary injury	(26–32)
Major depressive disorder (MDD)	1. Monoaminergic imbalance 2. Impaired synaptic plasticity 3. Neuroinflammation 4. Limited central drug delivery	1. Regulation of serotonin and dopamine signaling 2. Enhancement of synaptic plasticity 3. Reduction of inflammatory mediators 4. Transient BBB opening for targeted delivery	1. Improves depression-like behaviors 2. Supports circuit-level antidepressant intervention 3. Provides additional delivery potential	(33–38)

Nevertheless, substantial barriers must be addressed before LIPUS can be broadly translated into routine clinical practice. First, the field still lacks a standardized acoustic dose framework that can reliably link stimulation parameters to biological and therapeutic outcomes. At present, substantial heterogeneity exists across studies in carrier frequency, pulse repetition frequency, duty cycle, sonication duration, acoustic intensity, targeting strategy, and treatment schedule, which limits reproducibility and cross-study comparability. Future progress will require moving beyond empirical parameter selection toward a more rigorous dose–response model that integrates skull acoustics, tissue-specific energy deposition, target depth, and disease state.

Second, the mechanistic basis of LIPUS remains incompletely understood. Although mechanosensitive ion channels, calcium signaling, glial regulation, and vascular responses have all been implicated, the relative contribution of each pathway is likely to be context-dependent and disease-specific. A key future direction will be to identify the hierarchical relationships among these mechanisms and to determine whether LIPUS acts primarily at the level of neurons, glial cells, neurovascular units, or distributed brain networks under different conditions. In this regard, combining LIPUS with single-cell transcriptomics, *in vivo* calcium imaging, electrophysiology, and multimodal neuroimaging may help establish a more precise mechanistic atlas of ultrasound neuromodulation.

Third, current therapeutic models remain largely region-centered, whereas many neurological disorders are fundamentally disorders of distributed brain networks. This suggests that the next phase of LIPUS development should move from focal stimulation paradigms toward network-informed targeting strategies, in which disease-relevant circuits rather than isolated anatomical regions become the principal therapeutic targets. Such an approach may be especially important in disorders such as Alzheimer’s disease, epilepsy, and major depressive disorder, where pathological dysfunction is rarely confined to a single node.

Fourth, future translation may benefit from reconceptualizing LIPUS not only as a neuromodulation technology but also as a theranostic platform. Its ability to modulate neural activity, reshape inflammatory and vascular microenvironments, and in selected settings transiently open the blood–brain barrier creates opportunities for integration with pharmacotherapy, gene delivery, biologics, and rehabilitation interventions. This raises the possibility that LIPUS could serve as both a standalone intervention and an enabling technology that potentiates other treatments through spatiotemporally precise targeting.

Finally, robust clinical translation will depend on large-scale, well-controlled studies with long-term follow-up, as well as the development of sensitive biomarkers capable of capturing target engagement and therapeutic response. In this context, imaging-based readouts, electrophysiological signatures, circulating inflammatory markers, and digital behavioral phenotyping may together support a more precise and individualized therapeutic framework. As these challenges are progressively addressed, transcranial LIPUS may evolve from an experimental neuromodulation modality into a clinically actionable strategy for precision brain repair, thereby redefining therapeutic paradigms in neurological disease.
